# Adaptive intertemporal preferences in foraging-style environments

**DOI:** 10.3389/fnins.2013.00093

**Published:** 2013-06-17

**Authors:** Michael T. Bixter, Christian C. Luhmann

**Affiliations:** Department of Psychology, Stony Brook UniversityStony Brook, NY, USA

**Keywords:** intertemporal choice, foraging theory, optimality, dynamic decision-making, discounting

## Abstract

Decision makers often face choices between smaller more immediate rewards and larger more delayed rewards. For example, when foraging for food, animals must choose between actions that have varying costs (e.g., effort, duration, energy expenditure) and varying benefits (e.g., amount of food intake). The combination of these costs and benefits determine what optimal behavior is. In the present study, we employ a foraging-style task to study how humans make reward-based choices in response to the real-time constraints of a dynamic environment. On each trial participants were presented with two rewards that differed in magnitude and in the delay until their receipt. Because the experiment was of a fixed duration, maximizing earnings required decision makers to determine how to trade off the magnitude and the delay associated with the two rewards on each trial. To evaluate the extent to which participants could adapt to the decision environment, specific task characteristics were manipulated, including reward magnitudes (Experiment 1) and the delay between trials (Experiment 2). Each of these manipulations was designed to alter the pattern of choices made by an optimal decision maker. Several findings are of note. First, different choice strategies were observed with the manipulated environmental constraints. Second, despite contextually-appropriate shifts in behavior between conditions in each experiment, choice patterns deviated from theoretical optimality. In particular, the delays associated with the rewards did not exert a consistent influence on choices as required by exponential discounting. Third, decision makers nevertheless performed surprisingly well in all task environments with any deviations from strict optimality not having particularly deleterious effects on earnings. Taken together, these results suggest that human decision makers are capable of exhibiting intertemporal preferences that reflect a variety of environmental constraints.

Decision makers often face intertemporal choices: choices between actions associated with consequences that will be delivered at varying times in the future. For instance, many species often face the choice of consuming food immediately or caching it for future consumption. One central finding in the intertemporal choice literature is that both humans and other animals discount the value of rewards as a function of the delay until their delivery (e.g., Rachlin et al., 1991; Myerson et al., 2003; for reviews, see Frederick et al., 2002; Luhmann, 2009). In human intertemporal choice experiments, preferences are most often elicited by offering two rewards (usually monetary rewards), each associated with a different delay (e.g., $10 today or $25 in 3 months). After participants make a relatively large set of these intertemporal choices, experimenters typically estimate a discount function which represents the subjective value of future rewards (as a function of delay) for each individual decision maker.

The literature concerning intertemporal choice has been overwhelmingly concerned with the nature of the discount function that best describes discounting. One suggestion has been the exponential function, which is based on the continuously compounded utility function first proposed by Samuelson (1937). Exponential discounting can be formalized as follows:

(1) $$V_{D}=\frac{V_{0}}{e^{kD}}$$

where *V*_*D*_ is the discounted value of the delayed reward, *V*_0_ is the undiscounted value of the delayed reward (i.e., its objective magnitude), *k* is the discount rate that measures how quickly reward loses subjective value as a function of delay, and *D* is the length of delay until the reward's receipt. The exponential function is normatively attractive because it obeys the stationarity axiom (Koopmans, 1960). That is, when choosing between two rewards, the difference between the two delays will affect preferences, but a constant delay added to both rewards will not. This property also ensures dynamically consistent preferences (Strotz, 1955); if a decision maker prefers Reward_1_ to be delivered at Time_1_ over Reward_2_ to be delivered at Time_2_, this decision maker will never come to prefer Reward_2_ as time passes.

In contrast, empirical data from both humans (e.g., Myerson and Green, 1995; Kirby, 1997; Madden et al., 2003; but see Luhmann, 2013) and other animals (e.g., Rodriguez and Logue, 1988; Mazur, 2007) have led researchers to prefer the following hyperbolic function (Mazur, 1984, 1987):

(2) $$V_{D}=\frac{V_{0}}{1+kD}$$

where the parameters are the same those as defined in Equation 1. One property of this hyperbolic model is that regardless of the delay associated with a reward, each additional period of delay diminishes the discounted value by a smaller proportion (i.e., a diminishing decay rate). As a result, the preferences of a hyperbolic discounter are expected to change as the delay until a reward's receipt elapses (e.g., Thaler, 1981; Kirby and Herrnstein, 1995).

## Foraging-style paradigms

The study of human intertemporal choice has largely been driven by theories and empirical techniques derived from economics. For example, the intense focus on the descriptive accuracy of exponential and hyperbolic discounting functions is largely due to the conflict between economic prescriptions and empirical observations. Similarly, temporal preferences themselves tend to be evaluated using techniques (i.e., choices between pairs of delayed rewards) that are standard in economics. The overwhelming reliance on these types of decisions has not gone without critique (e.g., Hastie, 2001; Fawcett et al., 2012). A major theme in these critiques is a concern about how ecologically relevant these tasks are, because many real-world intertemporal dilemmas arise in dynamic environments where repeated, interdependent decisions are required to accomplish ultimate goals. Another potential limitation of most intertemporal choice tasks is that the costs associated with a choice (e.g., delay) are often minimally experienced by the participant. As a result, some researchers have developed experiential decision-making tasks (e.g., Herrnstein et al., 1993; Reynolds and Schiffbauer, 2004; Gureckis and Love, 2009; Luhmann et al., 2011) with the explicit goal of measuring temporal preferences in the face of actualized consequences.

Another reason to look for more naturalistic methods of studying intertemporal choice is because humans are not the only organisms that face intertemporal tradeoffs. Intertemporal tradeoffs are ubiquitous in natural environments, and are particularly evident in foraging decisions such as prey selection and patch exploitation/exploration. Optimal foraging theory (Pyke et al., 1977; Pyke, 1984; Stephens and Krebs, 1986) is a framework within behavioral ecology that characterizes foraging decisions as maximizing some currency (e.g., net energy gain or evolutionary fitness). Of particular interest within this framework has been the patch exploitation problem. In this scenario, foragers reside within a resource patch (e.g., a specific field of flowers) consuming the resources therein and thus depleting the patch as residence time increases. The exploitation problem refers to a persistent dilemma in which the forager must either stay within the current, ever less valuable patch or switch to a new resource patch that may or may not contain more resources. Intertemporal tradeoffs arise in these situations because there are a variety of delays affecting optimal behavior, including the travel time between patches, the average search time upon entering a patch and encountering a reward, and the handling time that elapses between encountering and experiencing a reward.

Studying decision making in more naturalistic environments is also important because doing so has the ability to reveal complexities in animal decision processes (including temporal preferences) that might differ from behavior observed in more contrived (e.g., laboratory) situations. For example, the study of non-human intertemporal choices are typically evaluated using an operant self-control paradigm, in which trials consist of a smaller-sooner reward (e.g., 1 pellet of food in 2 s) and a larger-later reward (e.g., 8 pellets of food in 10 s) being presented to the decision maker with the delay that follows each trial adjusted so that the total duration of a trial is the same regardless of which reward is chosen. The differences in temporal choice behavior between these self-control environments and foraging environments can be striking. For example, the behavior of pigeons (and other non-human animals) in such tasks has suggested that the subjective value of rewards drop 50% when delayed by just a few seconds (e.g., Green et al., 2004). In contrast, Stevens and Stephens (2008) describe how Clark's nutcracker birds routinely cache upwards of 30,000 seeds every autumn as insurance against the scarcity of subsequent winter months!

Patch exploitation tasks have only recently been used outside of ecology (see Sugrue et al., 2004; Adams et al., 2013). For instance, Hayden et al. (2011) had rhesus monkeys perform a virtual foraging task where choices were between a “stay” option which was analogous to the decision maker continuing to deplete a reward patch and a “switch” option which was analogous to the decision maker leaving the patch. Results indicated that certain behaviors of the rhesus monkeys conformed to predictions of optimal foraging models (the marginal value theorem specifically, Charnov, 1976). For example, as the length of the delay following a switch choice (i.e., travel time) increased, the monkeys chose the stay option more often (indicating longer patch residence time). This pattern is consistent with optimal foraging theory because these delays are analogous to travel times in natural environments (i.e., they represent the delay that elapses between switch choices and the next opportunity to earn rewards). Because rewards cannot be earned during these delays, they can be thought of as opportunity costs; the longer the delay, the more costly it is.

Adoption of patch exploitation paradigms has been even less common in the study of human intertemporal preferences (for recent exceptions, see Hutchinson et al., 2008; Kolling et al., 2012). What have become increasingly common in this field are tasks that incorporate critical aspects of traditional foraging paradigms. For example, there has been a recent surge of studies employing tasks in which choices influence both immediate rewards and the magnitude and/or availability of future rewards (e.g., Herrnstein et al., 1993; Gureckis and Love, 2009; Stillwell and Tunney, 2009; Otto et al., 2010). Other work has instead focused on choices made under time constraints. For example, Schweighofer et al. (2006) developed a repeated-choice task in which two reward items were presented on the screen on each trial (corresponding to a smaller sooner reward and a larger later reward). These items differed both in their magnitude and in the amount of time required to earn each reward. The entire task had a fixed time limit, which created a tradeoff between the magnitudes of the rewards available on each trial and the amount of time required to earn those rewards. As a result, participants were required to figure out the optimal strategy in order to maximize earnings. Schweighofer et al. (2006) found that human choices approximated optimality. Specifically, results suggested that participants did not discount rewards hyperbolically and instead behaved in a manner that closely approached exponential discounting. Given that standard intertemporal choice tasks have found that humans consistently discount hyperbolically, these results suggest that more naturalistic decision contexts can produce rational behavior in human decision makers (cf. Luhmann, 2013).

The goal of the present experiments was to further test the ability of human decision makers to make adaptive choices in foraging-style environments. The task developed by Schweighofer et al. (2006) was employed and various choice-relevant task parameters were manipulated. Specifically, to evaluate the extent to which participants could adapt to the decision environment, both reward magnitude (Experiment 1) and the delay between trials (Experiment 2) were manipulated. These manipulations were selected because optimal decision makers should respond to both manipulations by modulating their relative preferences for the larger, more delayed reward.

## Experiment 1

### Method

#### Participants

Forty-eight Stony Brook University undergraduates participated in exchange for partial course credit. One participant failed to sample one of the two choices and was thus excluded from further analyses. This left data from 47 participants in all of the following analyses. Twenty-three participants were included in the Large-Reward condition and 24 participants were included in the Small-Reward condition. The experiment was approved by the Committee on Research Involving Human Subjects (CORIHS) at Stony Brook University. Informed consent was obtained from all participants.

#### Materials and procedure

**Figure 1:**
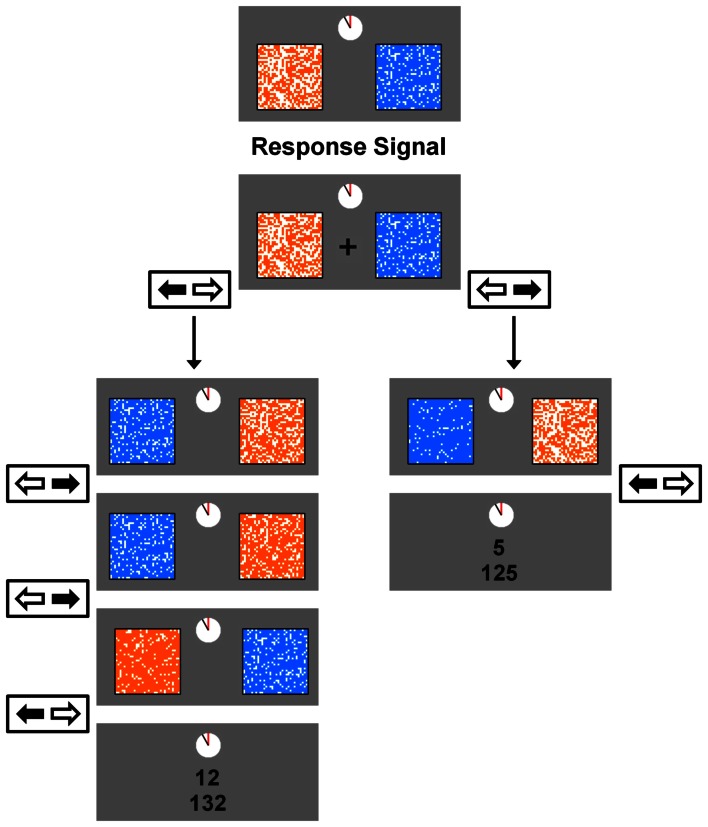
**An example trial in the experimental task.** On each trial, participants chose between an orange (the large reward) and blue (the small reward) grid. The number of filled cells in each grid represented the length of delay until that reward's delivery. A response signal (a fixation cross) appeared on the screen 500 ms after the reward pair was presented (the preview window), and remained on the screen for a minimum of 1 s (the response window) or until a participant made a response. Choices were made by pressing the left or right arrow key. On each step of a trial, choosing a reward filled an additional 1.5 s-worth of delay in that reward's grid. Once one of the grids was filled completely, the reward earned was displayed along with the total, accumulated rewards for the current block. The next trial began after the ITI, which was 1.5 s for both conditions in Experiment 1, 1 s for the Short-ITI condition in Experiment 2, and 6 s for the Long-ITI condition in Experiment 2. The reward magnitude for the large reward in this example illustrates the Small-Reward condition in Experiment 1. For the Large-Reward condition in Experiment 1 this magnitude was 24 points, and for both conditions in Experiment 2 it was 20 points.

***Task.*** A variation of the task used in the current experiments has been used and described before (e.g., Schweighofer et al., 2006, 2008; Tanaka et al., 2007). The task consisted of five blocks of trials, with each block lasting 210 s.^1^ On each trial, two reward items were presented on the screen (see Figure 1). Each reward item was represented by a 32 × 32 grid (1024 cells). One grid was blue and white (the smaller reward, *R*_*S*_) whereas the other grid was orange and white (the larger reward, *R*_*L*_). The number of white cells within the blue and orange grids represented the amount of time required to acquire the smaller reward (*D*_*S*_) and the larger reward (*D*_*L*_), respectively. The maximum delay employed in the task was 13 s, and was represented by a grid with 37 filled cells (4% of the grid). The minimum delay employed in the task was 0 s, and was represented by a grid with 987 filled cells (96% of the grid).

There were two between-subjects conditions, the Large-Reward condition and the Small-Reward condition. The values of *R*_*S*_ and *R*_*L*_ for the Large-Reward condition were 5 and 24 points, and for the Small-Reward condition they were 5 and 12 points. The values for all other task variables were the same between the two conditions. At the beginning of each trial, the smaller reward was associated with a new delay *D*_*S*_ (ranging from 1 to 6 s) and the larger reward was associated with a new delay *D*_*L*_ (ranging from 5 to 13 s). In order to acquire one of these rewards, participants had to repeatedly select a reward item over a series of steps until all the cells inside one of the two grids were filled. When one of the two reward items was chosen at each step in the trial, several unfilled cells within that reward item's grid were filled whereas the non-chosen reward item's grid remained the same. Specifically, 146 cells (14% of the grid) were filled after a choice, which corresponded to 1.5 s of delay (i.e., the length of a single step, see below) being subtracted from that reward item's total delay.

At the beginning of each step the newly revised delays were presented for 500 ms (the preview window), during which participants could not respond. A fixation cross then appeared at the center of the screen which signaled that participants could make their choice. This fixation cross remained on the screen for at least 1 s (the response window) or, in the event that no response was made within 1 s, until participants made a choice. Choices were made by pressing the left or right arrow key on the computer keyboard. The combination of the preview window and response window represented the 1.5 s step inter-stimulus-interval (Step-ISI) that occurred after a choice at each step. In order to increase attention toward the task, whether a reward item was presented on the left or right side of the screen was randomized at each step. Once one of the two rewards was earned, a 1.5 s inter-trial interval (ITI) occurred. During the ITI, the reward earned on the previous trial was displayed along with the total, accumulated rewards for the block displayed right below it.

Because the overall time limit associated with each block was critical, a clock-like figure was included at the top of the computer screen which represented how much time was left in the current block. This clock was present at all times throughout the task and the hand on the clock made one complete counter-clockwise revolution over the course of the block. Once this hand completed its revolution, participants completed the trial they were on and the block ended. After the completion of each block, participants were presented their total reward earnings for the block and told that they could press the spacebar to begin the next block of trials.

***Choice analysis***. Because the magnitudes of the larger and smaller rewards were fixed across the experiment, we formalized participants' choices as a function of the two delays presented on each trial. Specifically, Schweighofer et al. (2006) demonstrated analytically that optimal behavior in this task involved choosing the larger reward, P(L), with a probability proportional to

(3) $$-a_{L}D_{L}+D_{S}+a_{C}$$

where *D*_*S*_ and *D*_*L*_ are the delays associated with the smaller and larger rewards, respectively, on a given trial. This choice rule is analogous to assuming that the space of possible delay pairs (*D*_*S*_, *D*_*L*_) is bisected by an indifference line with slope $\frac{1}{a_{L}}$. If we define ω to be $\frac{1}{1+a_{L}}$ and γ to be *a*_*C*_(1−ω), Equation 3 suggests that choices should also be proportional to:

(4) $$\gamma +(1-\omega )D_{S}-\omega D_{L}$$

In this expression, it is more obvious that choices can be conceptualized as a function of a constant term, γ, and a weighted combination of the shorter and longer delays. Under Equation 4, the slope of the indifference line is $\frac{1-\omega }{\omega }$. Thus, ω = 0.5 corresponds to exponential discounting because preferences would only be sensitive to the difference between *D*_*L*_ and *D*_*S*_, thus obeying the axiom of stationarity (Koopmans, 1960). On a more intuitive level, when ω is 0.5, the decision maker is placing equal weight on both delays, whereas deviations from 0.5 indicate that the decision maker is placing more weight on *D*_*L*_ (ω > 0.5) or *D*_*S*_ (ω < 0.5). Schweighofer et al. (2006) found that humans in this task produced an average ω of 0.476, which closely approximates normatively preferred, exponential discounting.

Participants' preferences were characterized by fitting the following logistic regression model to participants' choices:

(5) $$P(L)=\frac{1}{1+e^{\left[-\rho \cdot [\gamma \;+\;(1\;-\;\omega )D_{S}\;-\;\omega D_{L}]\right]}}$$

where ρ is a stochasticity parameter that controls how deterministic a participant's choices are. Equation 5 was fitted separately for each individual participant by finding values for ρ, γ, and ω that maximized the likelihood of that participant's observed data. The only choice in a given trial that was used in all data analyses was the final choice of the trial sequence (i.e., the choice that led to one of the two reward items being received). Because it took multiple choices for a reward to be received on a given trial, it was possible that a participant's final choice in a trial did not match her initial choice at the beginning of the trial. However, for both conditions in both experiments, the proportion of trials where such reversals occurred was extremely small. For instance, in Experiment 1 it was less than 4% in both conditions, and in Experiment 2 it was less than 3% in both conditions.

Because the above choice rule bisects the choice space into two regions (i.e., the region in which the larger reward is preferred and the region in which the smaller reward is preferred), we were also able to compute an area-under-the-curve (AUC) measure. Specifically, AUC was the proportion of the space of possible *D*_*L*_/*D*_*S*_ pairs that would be expected to yield choices for the larger, delayed reward given the estimated choice parameters for that participant. Thus, these AUC values represented a relatively theory-agnostic measure of participants' preferences for the larger later rewards and allowed us to more directly evaluate whether the experimental manipulations had their predicted effects.

In order to find the values of γ and ω that would maximize earnings in each of the different conditions of Experiments 1 and 2 (i.e., the optimal parameter values), a search of the parameter space was performed with 5000 iterations of the task (as described in Table 1) being run to compute the expected earnings of each parameter pair. On each trial deterministic choices were made according to Equation 5 (i.e., choosing the larger reward when this quantity was positive, choosing the smaller reward when this quantity was negative). An initial grid search explored all integer values of γ between 2 and 10 and ω was allowed to vary between 0 and 1 in increments of 0.1. Values for γ and ω that jointly yielded the maximal payoffs were then used as the starting point for a second more granular search of the parameter space (using the Nelder-Mead simplex algorithm). The values for γ and ω produced by this procedure that maximized overall earnings within each condition were taken as optimal.

**Table 1:** **Values for reward, delay, and ITI variables in Experiment 1 and Experiment 2**.

**Variable**	**Experiment 1**	**Experiment 2**
*R*_*S*_	5	5
*R*_*L*_	**12 or 24**	20
*D*_*S*_	1–6 s	1–6 s
*D*_*L*_	5–13 s	5–13 s
Step-ISI	1.5 s	1.5 s
ITI	1.5 s	**1 or 6 s**
Block length	210 s	210 s

Note: See text for definitions of each variable. Values in bold represent the two conditions in each experiment.

### Results and discussion

**Figure 2:**
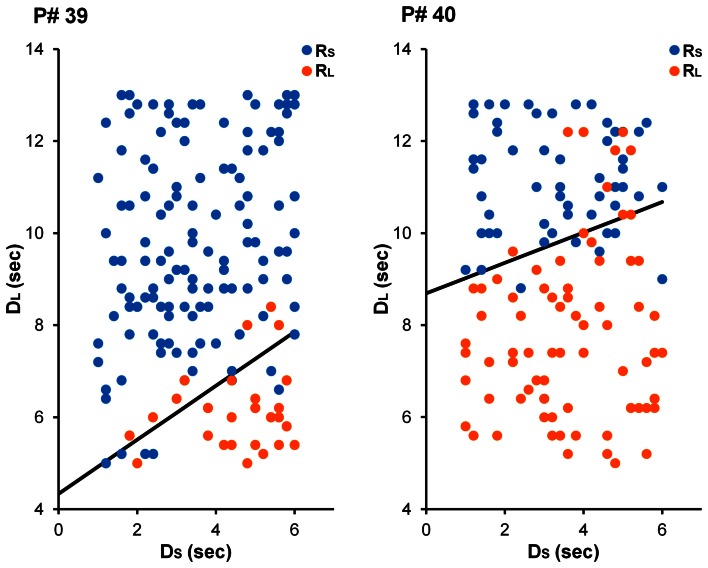
**Choices from illustrative participants in the Small-Reward (left) and Large-Reward (right) conditions in Experiment 1.** For the participant in the Small-Reward condition (participant #39), the best-fitting parameter estimates were γ = 2.73 and ω = 0.63. For the participant in the Large-Reward condition (participant #40), these estimates were γ = 6.53 and ω = 0.75.

**Figure 3:**
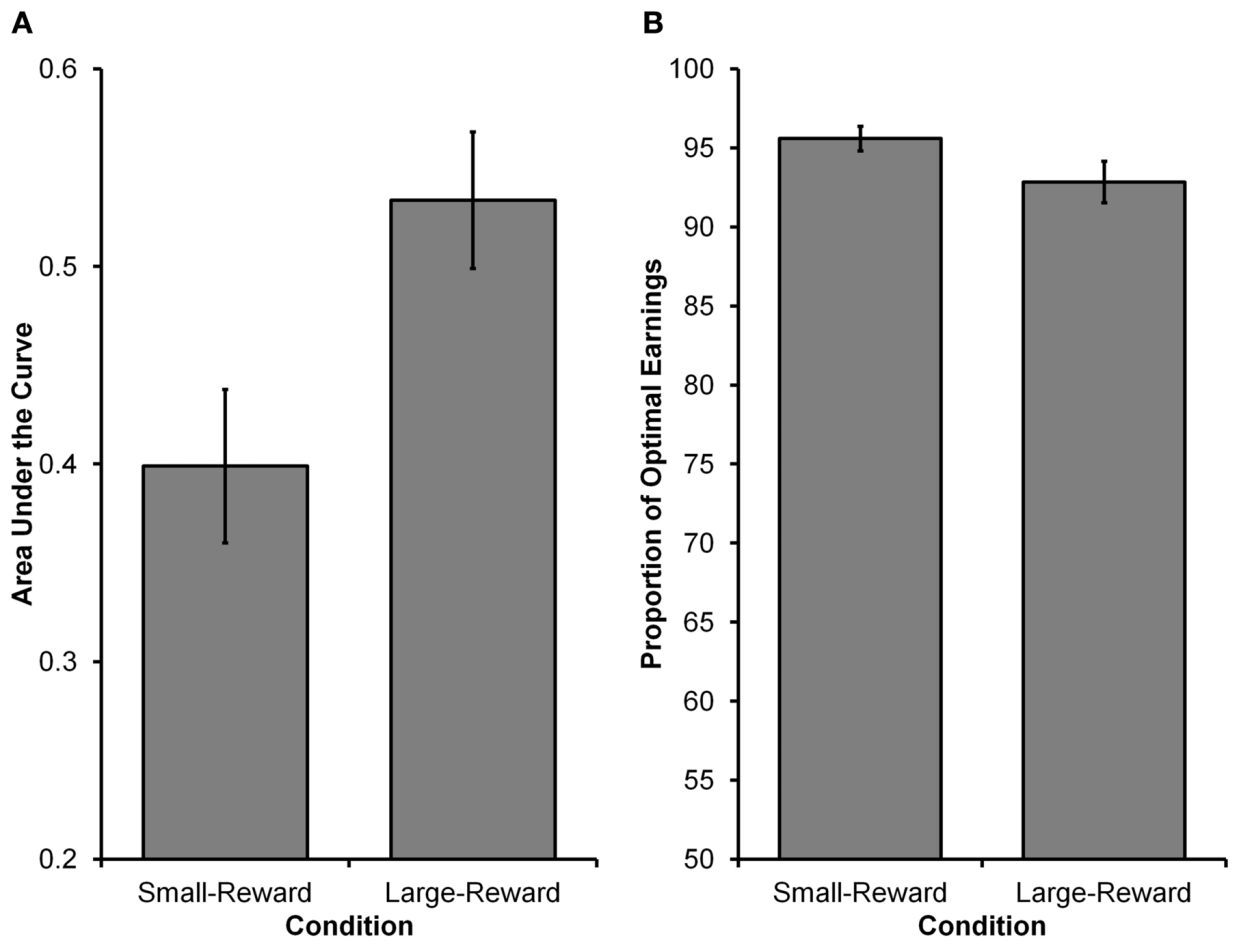
**Results from Experiment 1. (A)** The higher AUC value in the Large-Reward condition compared to the Small-Reward condition indicates a greater preference for the larger, more delayed reward (i.e., the orange grid). **(B)** The proportion of optimal earnings expected to be earned by participants based on their individual choice rules. Participants in both conditions were close to optimal, with participants in the Small-Reward condition having a marginally higher proportion than participants in the Large-Reward condition.

The main goal of Experiment 1 was to see whether decision makers were capable of modulating their intertemporal preferences in response to a manipulation of reward magnitude in a time-constrained environment. Specifically, participants in the Large-Reward condition were expected to exhibit a stronger preference for the larger reward than participants in the Small-Reward condition. See Figure 2 for choices from an illustrative participant in each condition. Equation 5 was fit to individual participants' choices and AUC was computed as a measure of intertemporal preferences (see Method section for details). As Figure 3A displays, participants in the Large-Reward condition had a higher AUC (*M* = 0.53, *SE* = 0.04) than participants in the Small-Reward condition (*M* = 0.40, *SE* = 0.04) [*t*_(45)_ = 2.58, *p* < 0.05]. This means that the manipulation had the predicted effect; increasing the difference between the magnitudes of the two rewards resulted in a stronger preference for the larger reward.

We next wanted to see how participants' choices in the two conditions were related to the choices of an optimal decision maker. For the Large-Reward and Small-Reward conditions, respectively, the optimal values for γ were estimated to be 4.10 and 2.98 and for ω they were both estimated to be 0.5. Observed parameter values for participants in the Large-Reward condition (*median* γ = 5.08, *SE* = 0.43; *median* ω = 0.64, *SE* = 0.04) and participants in the Small-Reward condition (*median* γ = 4.81, *SE* = 0.42; *median* ω = 0.69, *SE* = 0.03) deviated from the above optimal values. The most critical deviation from optimality was that participants did not weigh *D*_*S*_ and *D*_*L*_ equally (i.e., have a ω parameter of 0.5). Having ω values greater than 0.5 means participants were over-weighing *D*_*L*_ and under-weighing *D*_*S*_ (see the General Discussion for further discussion).

In order to determine how these deviations from optimality affected participants' performance in the task, we investigated the relationship between participants' expected earnings and the optimal earnings (see Method section for how optimal earnings were calculated). Calculating the expected earnings for an individual participant was found by using that participant's γ and ω estimates (i.e., the participant's choice rule) and used them in 5000 simulated runs of the experiment (assuming deterministic choices). As can be seen in Figure 3B, participants' expected earnings in both conditions were high in relation to optimal earnings. Participants in the Large-Reward condition were expected to earn 92.84% of the optimal earnings (*SE* = 1.30), whereas those in the Small-Reward condition were expected to earn 95.60% of the optimal earnings (*SE* = 0.78). The difference between the two conditions was marginally significant [*t*_(36.133)_ = 1.818, *p* = 0.077, corrected *df* due to unequal variances], indicating that participants' choices in the Small-Reward condition were earning slightly closer to optimal levels than participants' choices in the Large-Reward condition. However, participants in both conditions performed surprisingly well. Taken together, the details of participants' choice rules clearly deviated from optimality (i.e., they failed to place equal weight on each delay as expected under exponential discounting), but these deviations did not have particularly deleterious effects on earnings.

## Experiment 2

According to optimal foraging theory (specifically, the marginal value theorem, Charnov, 1976), choices in the patch exploitation paradigm should be directly influenced by travel time between patches. This is because longer travel times represent increased opportunity costs, which lead foragers to increase their preference for more fully depleting the current patch before switching to a new patch. Support for this prediction has been found across different species and foraging environments (e.g., Boivin et al., 2004). In contrast, many studies employing more traditional, laboratory intertemporal choice tasks have shown that the delay that elapses after the delivery of a reward (i.e., ITIs) has little or no influence on preferences (e.g., Mazur, 1989; Mazur and Romano, 1992; Bateson and Kacelnik, 1996).

Why the discrepancy between the self-control and foraging literatures? One explanation is that choices between mutually exclusive rewards in traditional laboratory intertemporal tasks and the sequential stay/switch choices common to patchy foraging environments are fundamentally different sorts of decisions (Stephens et al., 2004; Stephens, 2008; Stephens and Dunlap, 2009, 2011; Bourgeois-Gironde, 2012). For example, decision makers may view ITIs in traditional laboratory intertemporal choice tasks as irrelevant to reward earnings because all trials and rewards are independent (Pearson et al., 2010). In contrast, longer ITIs in sequential foraging situations (e.g., travel time) directly affect patch staying/switching decisions because of the opportunity costs they entail. That is, stay decisions allow the forager to continue to accrue rewards whereas switch decisions immediately halt the accrual of rewards (until travel to a new patch is complete) (Stephens and Anderson, 2001; Stephens and McLinn, 2003).

The goal of Experiment 2 was to investigate whether manipulation of the ITI duration in the current task might modulate participants' preferences. To get a sense of how such manipulations should affect choices in this task, imagine that a participant has some criterion which approximates the maximal value of *D*_*L*_ that a participant will tolerate in order to choose the larger reward on a given trial. Increasing the ITI should systematically increase this criterion. Why? Because selecting the smaller reward in order to advance to the next trial more quickly has now become a less valuable strategy. Opting for the smaller reward does end the current trial more quickly, but the decision maker must now face the cost of the longer ITI (e.g., reducing the amount of time left in the time-constrained task to earn future rewards) before being offered the next pair of rewards.

Experiment 2 seeks to test this prediction. Doing so will further evaluate the ability of humans to adapt to environmental constraints, because the delay that elapses between trials is likely a less salient facet of the task than the manipulations of reward magnitude performed in Experiment 1. Of course, because these factors are manipulated between-subjects, it is not possible for participants to compare the task environments. That being said, because of the intuitive relationship between reward magnitude and long-term earnings, the saliency (and relevance) of our reward manipulation may have been greater than the saliency of our ITI manipulation. As a result, Experiment 2 served as a stronger test of decision makers' sensitivity to the constraints created by a dynamic choice environment.

### Method

#### Participants

A new sample of 26 Stony Brook University undergraduates participated in exchange for partial course credit. Data from one participant was excluded because the participant was only run in one block of practice trials (as opposed to five). However, including this participant's data did not alter any of the patterns or levels of significance of the following results. This left 13 participants in the Long-ITI condition and 12 participants in the Short-ITI condition. The experiment was approved by the CORIHS at Stony Brook University. Informed consent was obtained from all participants.

#### Materials and procedure

The materials and procedure in Experiment 2 were the same as in Experiment 1, with the following exceptions. Because we were no longer interested in the effect of reward magnitude on choices, the reward amounts for the small and large rewards were fixed at five and 20 points, respectively, regardless of condition. However, the ITI that elapsed between the completion of one trial (i.e., the filling of one of the two grids) and the beginning of the next trial was systematically manipulated between conditions. For the Short-ITI and Long-ITI condition, the length of time that elapsed between trials was 1 s and 6 s, respectively.

### Results and discussion

**Figure 4:**
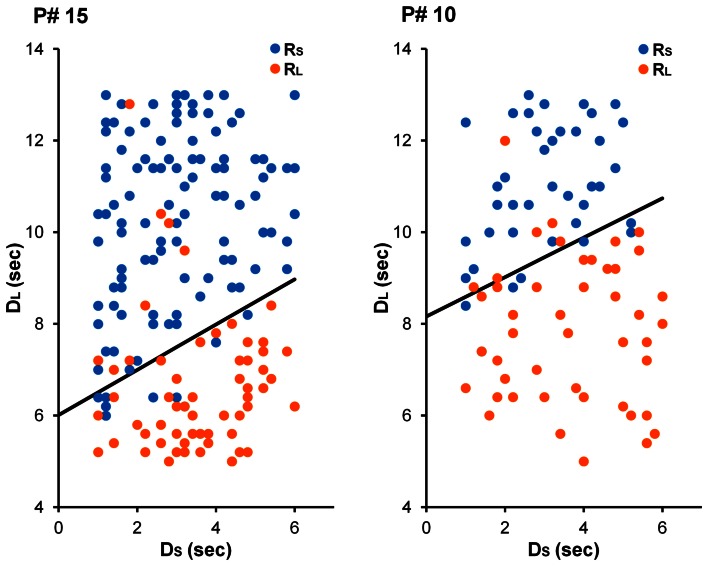
**Choices from illustrative participants in the Short-ITI (left) and Long-ITI (right) conditions in Experiment 2.** For the participant in the Short-ITI condition (participant #15), the best-fitting parameter estimates were γ = 4.02 and ω = 0.67. For the participant in the Long-ITI condition (participant #10), these estimates were γ = 5.71 and ω = 0.69.

**Figure 5:**
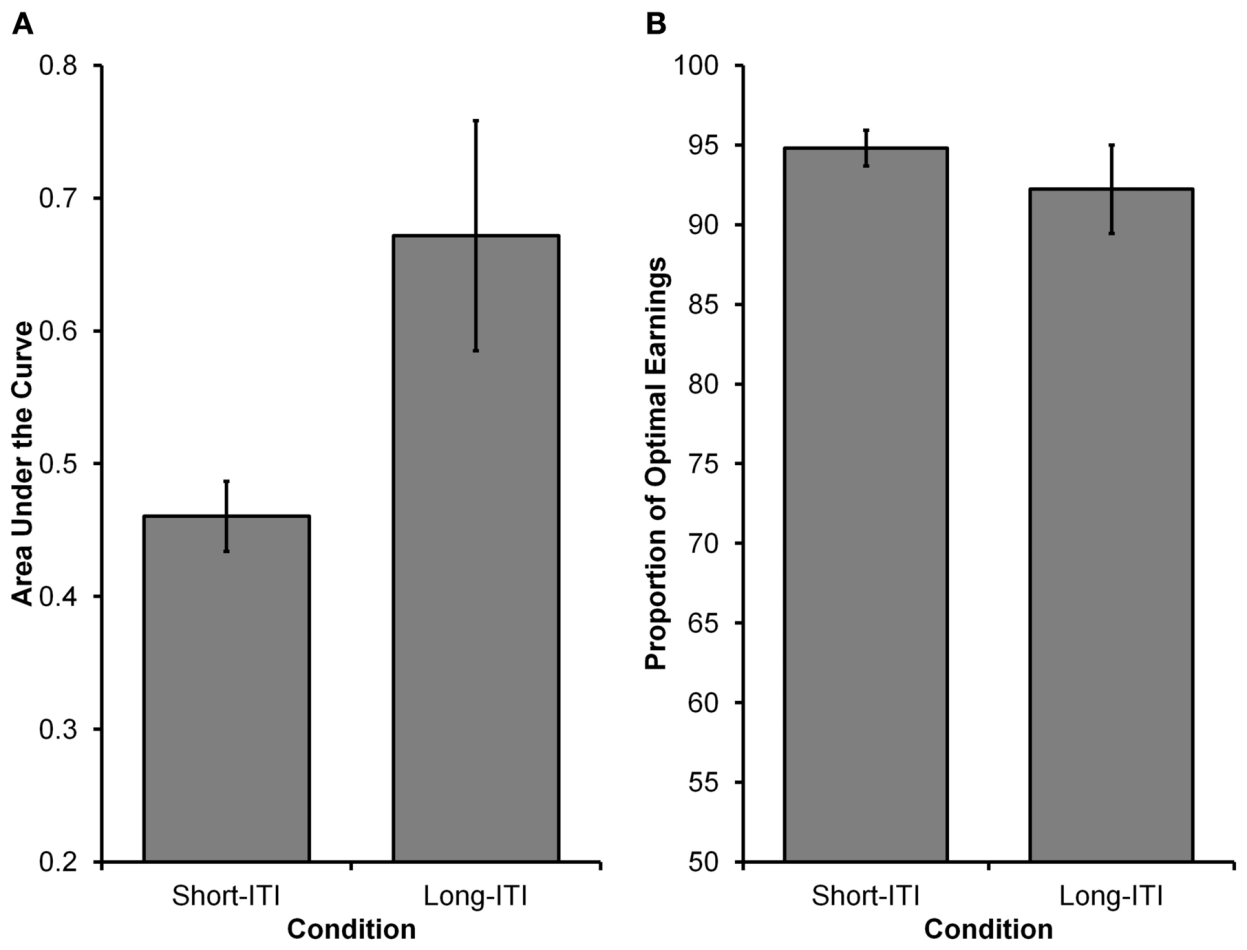
**Results from Experiment 2. (A)** The higher AUC value in the Long-ITI condition compared to the Short-ITI condition represents a greater preference for the larger, more delayed reward (i.e., the orange grid). **(B)** The proportion of optimal earnings expected to be earned by participants based on their individual choice rules. Participants in both conditions were close to optimal, with no differences observed between conditions.

See Figure 4 for choices from an illustrative participant in each condition. Similar to Experiment 1, Equation 5 was fit to participants' choices (see Experiment 1 Method section for details). As Figure 5A displays, it was found that participants in the Long-ITI condition had a higher AUC (*M* = 0.67, *SE* = 0.09) than participants in the Short-ITI condition (*M* = 0.46, *SE* = 0.03) [*t*_(14.209)_ = 2.334, *p* < 0.05, corrected *df* due to unequal variances]. This means that the manipulation had the predicted effect; increasing the ITI resulted in a stronger preference for the larger reward. So whereas it has been found that manipulations of ITI have little or no influence on intertemporal preferences in traditional laboratory self-control paradigms (e.g., Mazur, 1989; Bateson and Kacelnik, 1996), the current experiment shows that ITIs can have an influence on preferences when time-constraints are in place.

We again wanted to see how participants' choices in the two conditions related to the choices of an optimal decision maker. For the Long-ITI and Short-ITI conditions, respectively, the optimal values for γ were estimated to be 7.00 and 3.92 and for ω they were both estimated to be 0.5. Observed values for participants in the Long-ITI condition (*median* γ = 6.45, *SE* = 12.86; *median* ω = 0.68, *SE* = 0.31)^2^ and participants in the Short-ITI condition (*median* γ = 4.60, *SE* = 0.56; *median* ω = 0.67, *SE* = 0.05) deviated from the above optimal values. Once again, the most critical deviation from optimality was that participants did not weigh *D*_*S*_ and *D*_*L*_ equally (i.e., have a ω parameter of 0.5). Similar to Experiment 1, we investigated the relationship between participants' expected earnings and optimal earnings. In order to calculate the expected earnings for an individual participant, we used that participant's γ and ω estimates (i.e., the participant's choice rule) and used them in 5000 simulated runs of the experiment (assuming deterministic choices). As can be seen in Figure 5B, participants' expected earnings in both conditions were high in relation to optimal earnings. Participants in the Long-ITI condition were expected to earn 92.22% of the optimal earnings (*SE* = 2.78), whereas those in the Short-ITI condition were expected to earn 94.80 % of optimal earnings (*SE* = 1.12). The difference between the two conditions was not significant [*t*_(15.747)_ < 1, *p* > 0.40, corrected *df* due to unequal variances].

## General discussion

Most real-world decisions occur in dynamic environments where repeated choices are required to achieve long-term goals. Studying the ability of humans to adapt to various constraints in these types of environments can reveal elements of human decision making that are potentially more difficult to capture in standard intertemporal choice tasks. In the current study, each trial offered two reward items which differed both in their magnitude and in the amount of time required to earn them. Importantly, because the task had a fixed time limit, there existed a task-specific choice strategy that maximized reward earnings. Choice-relevant task parameters were manipulated in ways expected to influence the choice patterns of an optimal decision maker. These manipulations included expanding the difference in magnitude between the two rewards (Experiment 1) and increasing the delay between trials (Experiment 2). In each of these cases, the manipulations resulted in systematically different choice strategies and these differences were in the predicted directions. Taken together, these results suggest that participants are sensitive to environmental constraints and are capable of shifting their intertemporal preferences accordingly.

The current results further demonstrate the need to study intertemporal decision making using a variety of methodologies. As mentioned above, a predominant emphasis in the intertemporal choice literature has been on the pervasive “sub-optimality” of human and non-human decision making. For example, in laboratory self-control tasks where short- and long-term rewards are in conflict, it has been found that preferences are (1) dynamically inconsistent and (2) oftentimes “pathologically” impatient (Herrnstein and Prelec, 1991), meaning that the inability to delay gratification is detrimental to long-term welfare (e.g., Hausman, 1979; Herrnstein et al., 1993). Here we have used a foraging-style task in order to explore how human decision makers adapt their choice preferences under real-time constraints. The results of the current study as well as those of Schweighofer et al. (2006) demonstrate that human decision makers are able to adopt appropriate choice strategies, at least under certain circumstances.

Even though our participants' choices allowed them to earn a large percentage of the total rewards possible, there were systematic deviations from strict optimality that need to be noted. The most interesting of these is that, in all conditions in both experiments, participants put more weight on the delay associated with the larger reward (*D*_*L*_) than on the delay associated with the smaller reward (*D*_*S*_). This is an obvious deviation from the optimal pattern in which each of the two delays is given equal weight. One explanation for this deviation may be the fact that there was greater variability in *D*_*L*_ across trials. As a result, participants may have paid greater attention to this dimension and relatively less attention to the less variable *D*_*S*_. Alternatively, because *D*_*L*_ was associated with a larger, more desirable reward, participants may have focused on the delay that would be required to receive it to the relative exclusion of the delay associated with the smaller reward.

A more provocative explanation is that our subjects may not have been discounting the delayed rewards at all. For example, Scholten and Read (2010) have argued that many of the apparent deviations from economically normative standards (e.g., hyperbolic discounting) are not due to the shape of decision makers' discount functions, but actually a product of attribute-based intertemporal choices. That is, Scholten and Read (2010) suggest that a decision maker faced with a standard intertemporal choice (e.g., $10 in 4 days vs. $20 in 8 days) considers the difference between the reward magnitudes and the difference between the two delays. The ultimate decision about which delayed reward to select is then a function of a comparison between these two differences. Critical for the current results, Scholten and Read allow for the reward magnitudes, the delays, and the differences to be transformed and weighted. Thus, the over-weighing of *D*_*L*_ can be readily accounted for by their framework. This does not rationalize the over-weighing of *D*_*L*_ by participants in the current study—the attribute model is intended to be descriptive rather than normative—however it does provide a psychologically plausible account for intertemporal anomalies such as those observed in the current experiments.

Even though the task used in the current study simulates certain aspects of foraging environments, there are certain aspects that differ from most naturalistic patch decisions. For instance, in the current task, participants encounter two “patches” on each trial, even though patches are usually thought to be encountered sequentially. To more accurately simulate patchy environments, patches could be presented sequentially with reward intake being an increasing but decelerating function of patch residence time. In these more realistic situations, the duration of time participants spend in a patch should still be influenced by reward magnitude and ITI as in the current experiments. This prediction has been tested by Hayden et al. (2011) who did find that rhesus monkeys were sensitive to these sorts of manipulations. Research is needed to see if patch exploitation/exploration decisions in humans also conform to these predictions when making choices in resource-depleting patches. However, there are certain foraging situations, especially central place foraging situations (Houston and McNamara, 1985), where foragers leave a patch upon obtaining a single-item reward (e.g., diving seals returning to the surface after successfully obtaining a single prey item). The current task can be seen as a variation of this single-item reward situation, because each trial asked participants to select between “patches,” each of which led to a single-item reward and an associated interval of delay following the receipt of each reward.

The task used in the current experiments also deviated from natural foraging environments in that all rewards were delivered without any risk or uncertainty. This obviously conflicts with natural foraging environments where rewards are stochastic and uncertain (McNamara, 1982; Stephens and Krebs, 1986, Chapter 6; Nishimura, 1992; Kacelnik and Bateson, 1996, 1997; McDermott et al., 2008). Foraging risks can include predation risks, conspecific risks (e.g., food theft), and other collection risks which prevent the receipt of a reward over delay. To further increase ecological validity, foraging-style tasks with humans should go on to add risk constraints which have shown to influence foraging preferences in non-human animals, such as variance in the travel time (i.e., ITI) between patches (e.g., Kacelnik and Todd, 1992). Carrascal and Moreno (1993) found that the addition of risk (through greater presence of conspecifics) led to more immediate consumption and less caching in Nuthatch birds during foraging. The human intertemporal choice literature has also generally found that adding risk to larger, delayed rewards tends to shift preferences toward more immediate rewards (e.g., Mischel and Grusec, 1967; Keren and Roelofsma, 1995). However, it would be useful for future research to investigate how human decision makers respond to risk in foraging-style tasks that include intertemporal tradeoffs.

### Conflict of interest statement

The authors declare that the research was conducted in the absence of any commercial or financial relationships that could be construed as a potential conflict of interest.

## Footnotes

^1^Participants were also run in five practice blocks. After receiving instructions, participants were run in the first block of practice trials. If participants did not have any questions about the task, they were then run in four more blocks of practice trials to make sure they were familiar with the task.

^2^One participant's data in the Long-ITI condition was not well fit by the model. Without this participant, the Long-ITI condition has a standard error of 1.21 for the γ parameter and 0.08 for the ω parameter. Excluding this participant's data did not alter any of the reported results.
